# Age-Specific Incidence Data Indicate Four Mutations Are Required for Human Testicular Cancers

**DOI:** 10.1371/journal.pone.0025978

**Published:** 2011-10-06

**Authors:** James P. Brody

**Affiliations:** Department of Biomedical Engineering, Center for Complex Biological Systems, University of California Irvine, Irvine, California, United States of America; Ohio State University Medical Center, United States of America

## Abstract

Normal human cells require a series of genetic alterations to undergo malignant transformation. Direct sequencing of human tumors has identified hundreds of mutations in tumors, but many of these are thought to be unnecessary and a result of, rather than a cause of, the tumor. The exact number of mutations to transform a normal human cell into a tumor cell is unknown. Here I show that male gonadal germ cell tumors, the most common form of testicular cancers, occur after four mutations. I infer this by constructing a mathematical model based upon the multi-hit hypothesis and comparing it to the age-specific incidence data. This result is consistent with the multi-hit hypothesis, and implies that these cancers are genetically or epigenetically predetermined at birth or an early age.

## Introduction

Tumors originate from a single cell after the cell accumulates a series of mutations [1]–[6], according to the multi-hit model of cancer [2], [3]. These mutations can include many different types of alterations to the DNA including methylation, single base substitutions, and duplications or deletions of chromosomes. The exact number of mutations required to transform a normal human cell into a tumor cell is unknown [7].

Direct DNA sequencing of tumors has established an upper limit on the number of mutations required to transform a cell. Sequencing of breast and colorectal cancers identified about 80 mutations in a typical tumor [8]. Further statistical analysis suggested that less than 15 of those 80 are necessary [8]. A second experiment sequenced 623 known cancer-related genes in a set of 188 lung adenocarcinomas showing more than 1000 somatic mutations. Further analysis identified 26 genes that were concluded to be involved in carcinogenesis [9].

A lower limit on the number of mutations to transform a normal cell has been established in the laboratory. A human tumor cell was synthesized from normal human cells (both epithelial and fibroblast cells) by altering the expression of only three genes, which effected four biochemical pathways [6], [10]. This tumor cell displayed the classic characteristics of a human tumor cell: anchorage-independent growth and formation of tumors in nude mice.

It is widely believed that colon tumors require four to six mutations [11]. This is based upon comparing the Armitage and Doll equation, 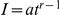 [12], to age-specific incidence data. But many problems exist with this [13]: it fails to describe the data at older ages; it increases without any upper limits; and it does not incorporate clonal expansion.

Testicular gonadal germ cell cancers differ from other solid tumors in a number of ways. First, the incidence of gonadal germ cell tumors is highest at about 30 years of age and declines to just a handful of cases diagnosed in men in their 70's. In comparison, the incidence of many other solid tumors increases with age. Second, combination chemotherapy is particularly effective against testicular gonadal germ cell tumors as compared to other solid tumors. Finally, most solid tumors originate in somatic cells, while most testicular cancers arise in germ cells.

The cause of testicular gonadal germ cell tumors is not known [14], [15]. No known environmental factors affect its development [16]. A family link exists, stronger in brothers than father and sons [17]. Testicular cancers showed the third highest heritability, but most cases are sporadic [18]. Age standardized rates of testicular cancer have increased over the past few decades in the United States [19] and in other parts of the world [20].

The strongest association of testicular cancer with any other medical conditions is with cryptorchidism, where the testicles do not descend into the scrotum at birth. About 5 to 10% of those who develop testicular cancer had undescended testicles at birth, compared to about 2 to 5% in the general population [21]. It is not known whether cryptorchidism causes testicular cancers or whether both are caused by a common factor.

Two hypotheses exist for the origin of testicular cancers. The first suggests that testicular cancers are determined *in utero* or at an early age [22], [23]. A second is that environmental exposure to carcinogens throughout ones lifetime leads to the development of a tumor, while genetics modifies this environmental risk [24]. Although this second hypothesis is widely believed, little evidence exists that environmental mutagens cause any of the point mutations observed in human cancers [25].

Tests of these hypotheses are mixed. A retrospective study of Swedish males found that those who underwent surgery before the age of 13 to correct undescended testicles had a slightly lower risk of developing testicular cancer than those who did not undergo surgery [21]. This suggests that testicular cancers could not be predetermined at birth. However, a similar study containing almost twice as many subjects in Denmark found no significant change in the incidence of testicular cancer after surgery for undescended testicles [26].

Two genome wide association studies identified several mutations that predispose to the development of testicular tumors [27], [28]. These mutations are located in two genes, *KITLG* and *SPRY4*, that are known to play a role in testicular development. The estimated per allele odds ratio for these are among the highest found for any genome wide association study of a cancer [29].

Previously, others have sought to understand the age-specific incidence of cancers with different approaches. Including using a Weibull distribution for lung cancer [30], analyzing the age-specific acceleration of cancers [31], [32], modifying the Armitage Doll equation directly with a damping term [33]–[35], and using a multistage model with age-dependent behavior to estimate the number of mutations required to develop breast cancer [36]. An analysis of Danish and Norwegian cancer registries suggests that testicular cancer age-specific incidence data are best modeled with a frailty effect, where a portion of the population is non-susceptible to developing the cancer [37].

The objective of this paper is to determine how many mutations are required to develop testicular cancer. The approach is to compare the expected age-specific incidence, based upon the multi-hit model, with the measured age-specific incidence for testicular cancers.

## Results

**Figure 1:**
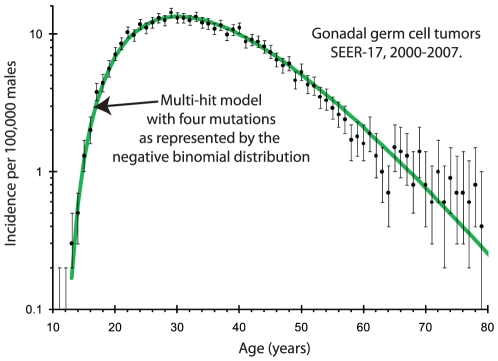
This is a comparison between the observed (SEER-17, 2000–2007) age-specific incidence for testicular cancers and that predicted by the multi-hit model with four mutations. The black circles represent the measured incidence, the error bars are 95% confidence intervals, and the green solid line represents the incidence predicted by the multi-hit model with four mutations.

The age-specific incidence for testicular cancers is accurately described by Equation (1) with four mutations. Figure 1 shows a comparison between Equation (1) and data for all eight years. This also implies that testicular cancers develop from a single progenitor cell. Table 1 shows the parameters and error estimates, and p-value for individual years.

**Table 1:** The best estimate of the parameters 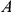 (number of men per 100,000 who will ultimately develop testicular cancer in their lifetime) and 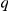 (the probability per year that no mutation occurs).

Year	P-value	A	q
2000	0.017	387	0.856
2001	0.043	381	0.856
2002	0.059	379	0.855
2003	0.497	370	0.854
2004	0.316	387	0.851
2005	0.004	381	0.851
2006	0.722	382	0.854
2007	0.350	376	0.846
	0.2606	380( 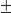 5)	0.853( 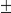 0.003)

I tested the hypothesis that the testicular cancer age-specific incidence data were derived from Equation 1. To obtain the best estimate of the parameters in Equation 1, I performed a least squares fit of Equation 1 to the age-specific incidence data for eight consecutive years (2000–2007). This table lists the best estimate of the parameters 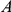 (number of men per 100,000 who will ultimately develop testicular cancer in their lifetime) and 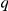 (the probability per year that no mutation occurs). It also lists the p-value, the probability that the hypothesis should not be rejected.

**Figure 2:**
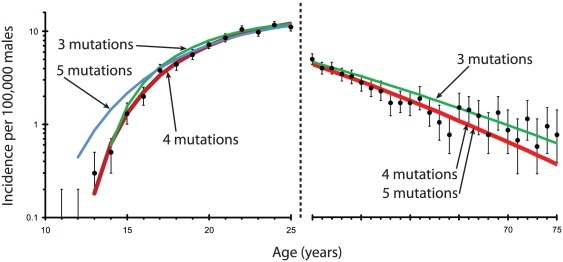
This figure compares the best fit models for three, four, and five mutations. To emphasize the differences, only regions before the age of 25 and after the age of 50 are shown. All three models show close fit to the data between 25 and 50 years of age. The models with four mutations and five mutations are identical after the age of 50.

Figure 2 compares the best fits for models with three, four, and five mutations. The model with four mutations was the best fit. Three mutations provided a slightly worse fit in the 15–20 and 65–75 age range, while five mutations provided a much worse fit in the range of 10–25 years, but was indistinguishable from 4 mutations in the 50–75 age range.

I measured the probability of advancing to the next stage at 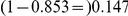 per year. This measures only the probability of a mutation that would advance the precancerous tissue another step towards cancer, and is not directly comparable to measured mutation rates. Human germ line mutations vary across the genome by orders of magnitude [38]; a single mutation rate cannot accurately characterize the process.

## Discussion

The approach presented here implicitly assumes that the probability of advancing to the next stage, which could be associated with a mutation rate, is constant. Complexity could be added to the model by modifying this assumption. At least two different mechanisms could modify the mutation rate in a pre cancerous tissue. First, the mutator phenotype hypothesis suggests that one of the first mutations on the path to a tumor must result in a higher mutation rate [39]. Second, the process of clonal expansion can expand the pool of cells at one stage, increasing the probability of advancing to the next stage [40]–[42]. Since the simplest assumption, a constant probability of advancing, was sufficient in this case, I did not extend the model to include a changing probability rate.

The mutations are most likely chromosomal additions or deletions, not single base alterations. Cytogenetic studies of seminoma and non-seminoma testicular cancers have shown consistent alterations to several chromosomes. In particular, amplification of a region of chromosome 12p containing several known genes is often present [43].

Although four mutations are required for the development of testicular cancers, these mutations may alter more than four genes and biochemical pathways. In addition, other mutations that are not rate limiting may occur. Non rate limiting mutations would not alter the age-specific incidence data.

One potential problem with this analysis is that it assumes no significant long term change in the rate of testicular cancers. The SEER-9 data show that the age-adjusted testicular cancer rate has increased by about 7% per year from 1973 to 2008. The standard way for dealing with temporal variation in cancer rates is to first analyze age models, then age plus drift, then age-period and/or age-cohort, and finally age-period-cohort models [44], [45]. Each addition of complexity requires additional parameters and reduces the number of degrees of freedom. Since the age only model provided a good fit to the data, further complexity was avoided. However, future work on the age-specific incidence of testicular cancer should explore whether these more complex models provide alternative solutions.

Additional complexity to the model could be added in different ways. To account for inherited mutations, the model could consist of two independent terms similar to Equation (1). The first term would require 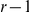 mutations and the second term would require 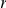 mutations. To account for multiple pathways by which testicular cancer could develop, the model could be extended by adding a second term with independent parameters from the first. Neither of these additions are necessary, but the data does not exclude the possibility of these more complex processes.

The agreement between the age-specific incidence data and Equation (1) implies that testicular cancers have a single potential progenitor cell. This contrasts with most other types of solid tumors which are thought to have many, many potential progenitor cells.

The age-specific incidence data implies that testicular cancers are pre-determined before the age of 10 and possibly at birth either through genetic or epigenetic [46] predisposition. This data is inconsistent with a hypothesis where exposure to environmental carcinogens in mature men lead to the development of a testicular tumor.

## Methods

The multi-hit model describes a series of independent Bernoulli trials. A random number is drawn between zero and one. If the number is less than 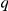, no mutation occurs; if greater than 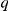, a mutation occurs. The process is repeated periodically. When 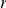 mutations have occurred, a tumor begins to develop. The tumor grows, through clonal expansion, over an additional time 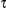 until it is detected as a cancer. The time 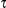 might also be related to normal growth and development. This process occurs in a fraction of the population, 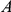, that lies somewhere between 0 and 100%.

Under these assumptions, the probability distribution for the age at which testicular cancer is diagnosed should be given by the solution to the series of independent Bernoulli trials, the negative binomial distribution [47]

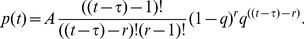
(1)The age-specific incidence measures the hazard function, which is related to Equation (1) by dividing by the ratio of the total population to the at-risk population. Males who have been previously diagnosed with testicular cancer are removed from the total population to produce the at-risk population. The effect of this is at most 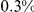 on 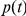 and can be ignored in this case since it is overwhelmed by the predominant sampling error.

One assumption in the derivation of Equation (1) is that a single progenitor cell exists in the tissue. If many progenitor cells exist, as is thought to occur in most tissues, then cancer is diagnosed when the first of these many cells develops into a tumor. In this case, the first order statistic, or distribution of the minimum, of Equation (1) is the proper equation to compare to the age-specific incidence data. This would follow a Weibull distribution [30], [48].

I tested the hypothesis that the age-specific incidence data on testicular germ cell tumors is accurately described by Equation (1). I performed a least squares fit to determine the parameters of the equation for all eight years in the dataset. Then, I calculated the reduced chi-squared value and the associated p-value, given the number of degrees of freedom, 53. The p-value, shown in Table 1, indicates the probability that the hypothesis should not be rejected.

In the United States, the Surveillance, Epidemiology, and End Results (SEER) Program of the National Cancer Institute (NCI) collects data on cancer cases. It is considered the gold-standard for data quality for cancer registries. It collects data from 17 different geographic regions that encompass just over 26% of the population of the United States [49]. This data is combined with US Census data on the population, as a function of age, in the these 17 geographic areas to calculate the age-specific incidence.

I obtained testicular germ cell tumor age-specific incidence data using SEER*Stat (version 6.6.2) [50]. SEER*Stat allows one to easily query the SEER case files. I queried the database published in November 2009, the SEER 17 incidence database with single ages to 85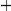 [49]. This was the most recent available. I selected all reported tumors that were in males, located in the testis, and classified as germ cell or trophoblastic tumors or neoplasms of gonads, totaling 16,291 cases. I excluded testicular cancers diagnosed before the age of 13, because they were probably due to a different mechanism, 88 cases were excluded. Those diagnosed before the age of four are probably teratoma-yolk sac tumors [43]. These account for only a small fraction (0.5%) of all testicular cancers. (From 2000–2007, the SEER-17 registries recorded 88 testicular germ cell cancers in patients under 13, 72 of these cancers were diagnosed in the first 36 months of life.)

I compared Equation (1) to age-specific incidence data collected by the SEER-17 cancer registries from 2000–2007 on the incidence of testicular cancers, both seminomas and non-seminomas. I compared these equations with the data from ages 13 to 70 years old and with the number of mutations, 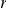, ranging from three to five. This comparison was made by minimizing the reduced chi-squared value [51] using the Generalized Reduced Gradient algorithm. This algorithm is suitable for minimizing non-linear functions. I used multiple starting points to ensure that the solution given was the global minimum and not a local minima.

I calculated error estimates by measuring the parameters 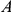 and 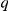 for eight individual years (2000–2007) and taking the standard deviation of these eight values. Uncertainty in the parameter, 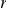, which represents the number of mutations required to develop a tumor, was measured by comparing models for three, four, and five mutations, as shown in Table 2.

**Table 2:** The best fit parameters for each model, along with the calculated P-value.

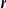	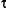	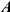	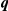	P-value
3	11	386	0.884	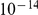
4	9	377	0.851	0.02
5	5	378	0.8388	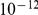

I tested models with 3, 4, and 5 rate-limiting steps fitting these models to combined 2000–2007 testicular cancer data. The best fit parameters for each model, along with the calculated P-value, are shown here.
